# The Centrioles, Centrosomes, Basal Bodies, and Cilia of *Drosophila melanogaster*

**DOI:** 10.1534/genetics.116.198168

**Published:** 2017-05-03

**Authors:** Ramona Lattao, Levente Kovács, David M. Glover

**Affiliations:** Department of Genetics, University of Cambridge, CB2 3EH, United Kingdom

**Keywords:** *Drosophila*, basal body, centriole, centrosome, cilia, FlyBook

## Abstract

Centrioles play a key role in the development of the fly. They are needed for the correct formation of centrosomes, the organelles at the poles of the spindle that can persist as microtubule organizing centers (MTOCs) into interphase. The ability to nucleate cytoplasmic microtubules (MTs) is a property of the surrounding pericentriolar material (PCM). The centriole has a dual life, existing not only as the core of the centrosome but also as the basal body, the structure that templates the formation of cilia and flagellae. Thus the structure and functions of the centriole, the centrosome, and the basal body have an impact upon many aspects of development and physiology that can readily be modeled in *Drosophila*. Centrosomes are essential to give organization to the rapidly increasing numbers of nuclei in the syncytial embryo and for the spatially precise execution of cell division in numerous tissues, particularly during male meiosis. Although mitotic cell cycles can take place in the absence of centrosomes, this is an error-prone process that opens up the fly to developmental defects and the potential of tumor formation. Here, we review the structure and functions of the centriole, the centrosome, and the basal body in different tissues and cultured cells of *Drosophila melanogaster*, highlighting their contributions to different aspects of development and cell division.

CENTROSOMES are the organelles at the poles of the spindle that can persist into interphase as microtubule organizing centers (MTOCs). At their core is a ninefold symmetrical centriole, and their ability to nucleate cytoplasmic microtubules (MTs) is a property of the surrounding pericentriolar material (PCM; Figure 1). The centriole has a dual life, existing not only as the core of the centrosome but also as the basal body, the structure that templates the formation of cilia and flagellae. As a result, the structure and function of the centriole, the centrosome, and the basal body have an impact upon many aspects of development and physiology, resulting in diseases that can readily be modeled in *Drosophila*. Such diseases include many forms of cancer, where centrosome aberrations have been known for over a century, and a variety of heritable diseases, where developmental disorders characterized by defects in cilia (ciliopathies) or asymmetric cell divisions in the brain (microcephaly) are associated with cilia, centriole, and centrosome defects.

**Figure 1:**
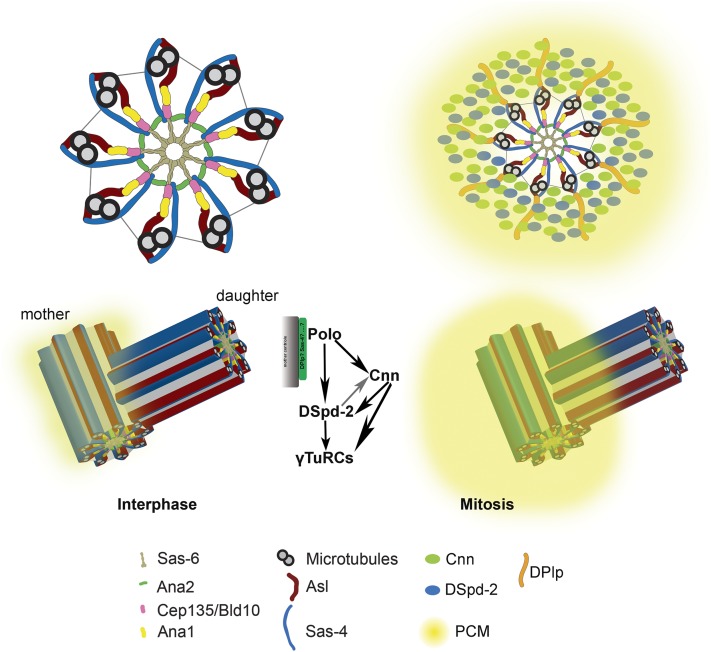
Organization of the centrosome. Originally discovered by van Benenden and Boveri at the end of the 19th century as a body that increased its size at the spindle poles during mitosis, the centrosome first revealed its structural secrets to electron microscopy. Here, we lay 21st century molecular detail onto the ninefold symmetry of the centriole and its surrounding PCM that increases in amount during mitosis, shown in section (above) and in 3D (below). The diagram show the components involved in PCM recruitment around the mother centriole. See text for details of the molecular components, which are not shown to scale. PCM, pericentriolar material.

Studies of centriole biology initially depended upon the application of electron microscopy (EM) focused upon the roles of centrioles in critical stages of development. This led, for example, to the discovery that once the nurse cells of the egg chamber begin their endoreduplication cycles, the centrioles migrate to accumulate at the oocyte before ultimately being eliminated so that female meiosis can occur on a centriole-free spindle (Mahowald and Strassheim 1970). EM also gave us the first detailed description of the complex behavior of the centriole in the male germline (Tates 1971), which we will discuss further below. However, progress was greatly accelerated when antibodies became available that recognized centrosomal components, so permitting centrosome behavior to be tracked using immunofluorescence microscopy. Curiously, one of the first centrosomal antigens to be followed in this way, CP190, first identified through a monoclonal antibody, Bx63 (Frasch *et al.* 1986), may not have its major functions at the centrosome. The Bx63 antigen was later purified as a MT-associated protein (MAP) (Kellogg and Alberts 1992) and indeed does have a MT-binding domain and some function in spindle assembly (Plevock *et al.* 2015). However, its major role is as a chromatin insulator that is critical to direct the formation of chromosomal loops to organize transcription units in the interphase nucleus [reviewed by Ahanger *et al.* (2013)]. This highlights the difficulty of many early studies of centrosome proteins to know whether they were taking a “joy ride” on the centrosome or its associated MTs or that they really functioned there (Table 1). Irrespective of its function, CP190 proved to be an invaluable antigen with which to trace centrosome behavior in early cell biological studies, particularly in the rapid nuclear division cycles of the syncytial embryo.

**Table 1:** List of proteins discussed in the text

Protein	Human homolog	Functions	References
Ana1	CEP295	Centriole duplication, centriole-to-centrosome conversion, centriole length control	Blachon *et al.* (2009); Fu *et al.* (2016); Saurya *et al.* (2016)
Ana2	STIL	Centriole assembly, Sas-6 recruitment	Stevens *et al.* (2010a); Dzhindzhev *et al.* (2014); Cottee *et al.* (2015)
Aurora	AURORA A	Kinase, cell cycle regulator	Leibowitz (1990); Glover *et al.* (1995); Giet and Glover (2001); Giet *et al.* (2002)
Asl	CEP152	Centriole duplication, Plk4 recruitment and stabilization, centriole-to-centrosome conversion, centriole length control, flagellar axoneme	Dzhindzhev *et al.* (2010); Novak *et al.* (2014); Klebba *et al.* (2015); Fu *et al.* (2016); Galletta *et al.* (2016a)
Bld10/Cep135	CEP135	Centriole elongation and stability, centriole-to-centrosome conversion, centrosome asymmetry in NBs	Matsuura *et al.* (2004); Hiraki *et al.* (2007); Carvalho-Santos *et al.* (2012); Roque *et al.* (2012); Lerit and Rusan (2013); Singh *et al.* (2014); Fu *et al.* (2016)
Bsg25D	Ninein	Centrosome asymmetry in NBs?	Zheng *et al.* (2016)
Bug22	GTL3/CFAP20/hBug22	Centriole length control	Mendes Maia *et al.* (2014)
Centrobin	CENTROBIN	Centrosome asymmetry in NBs, Proximal daughter centriole in chordotonal organs	Januschke *et al.* (2013); Galletta *et al.* (2014); Gottardo *et al.* (2015a)
Cep290	CEP290	Cilia assembly	Basiri *et al.* (2014)
Chibby	CHIBBY	Cilia assembly	Enjolras *et al.* (2012); Basiri *et al.* (2014); Pratt *et al.* (2016); Vieillard *et al.* (2016)
Cnn	CDK5RAP2	PCM recruitment and organization	Conduit *et al.* (2014b); Lerit *et al.* (2015); Eisman *et al.* (2016)
CP110	CP110	Centriole length control	Schmidt *et al.* (2009); Franz *et al.* (2013)
CP190	????	Chromatin insulator, MAP	Frasch *et al.* (1986); Kellogg and Alberts (1992); Ahanger *et al.* (2013); Plevock *et al.* (2015)
Dilatatory	CEP131	Cilia assembly	Basiri *et al.* (2014)
DPlp	PCNT	PCM recruitment and organization, centrosome asymmetry in NBs, marks the mother centriole or basal body in chordontonal organs	Megraw *et al.* (2002); Martinez-Campos *et al.* (2004); Mennella *et al.* (2012); Galletta *et al.* (2014); Lerit *et al.* (2015); Ramdas Nair *et al.* (2016)
Spd-2	CEP192	PCM recruitment and organization, APC/C recruitment to the interphase centriole in NBs	Dix and Raff (2007); Giansanti *et al.* (2008); Meghini *et al.* (2016)
DTACC	TACC3	Microtubule stabilization	Barros *et al.* (2005)
Klp10A	KIF2A?	Kinesin-like protein, centriole length control	Delgehyr *et al.* (2012)
MKS complex	MKS complex	Cilia assembly	Basiri *et al.* (2014); Vieillard *et al.* (2016)
Msps	XMAP215	Microtubules stabilization	Barros *et al.* (2005)
Plk4	PLK4	Kinase, centriole duplication, PCL formation	Bettencourt-Dias *et al.* (2004), (2005); Rodrigues-Martins *et al.* (2007b); Blachon *et al.* (2009); Cunha-Ferreira *et al.* (2009), (2013); Rogers *et al.* (2009); Brownlee *et al.* (2011); Lopes *et al.* (2015); Saurya *et al.* (2016)
Poc1	POC1	Centriole length control, PCL formation	Blachon *et al.* (2009)
Polo	PLK1	Kinase, cell cycle regulator, centriole disengagement, PCM recruitment, centrosome asymmetry in NBs, centriole loss during oogenesis	Sunkel and Glover (1988); Llamazares *et al.* (1991); Fenton and Glover (1993); Rusan and Peifer (2007); Pimenta-Marques *et al.* (2016)
Rootletin	ROOTLETIN	Rootlet formation	Chen *et al.* (2015); Styczynska-Soczka and Jarman (2015)
Sas-4	CPAP	Centriole assembly and elongation	Basto *et al.* (2006); Hsu *et al.* (2008); Gopalakrishnan *et al.* (2010); Tang *et al.* (2011); Conduit *et al.* (2015a)
Sas-6	SAS-6	Establishment of centriolar ninefold symmetry, PCL formation	Blachon *et al.* (2009); Gopalakrishnan *et al.* (2011); Kitagawa *et al.* (2011); Van Breugel *et al.* (2011), (2014); Cottee *et al.* (2015)
Uncoordinated	NA	Cilia assembly	Baker *et al.* (2004); Enjolras *et al.* (2012); Vieillard *et al.* (2016)
Wdr62	MCPH2	Microtubule stabilization	Ramdas Nair *et al.* (2016)

NB, neuroblasts; PCM, pericentriolar matrix; MAP, microtubule-associated protein; APC/C, anaphase-promoting complex/cyclosome; PCL, proximal centriole-like; NA, not applicable.

## The Parts Catalog

### Taking advantage of the fly’s life cycle

The identification of key components of the centrosome has been critical to understanding its duplication and function. As a major function of the *Drosophila* centrosome is as a MTOC in cell division, and because the organelle is essential for syncytial embryo development and male fertility, it is perhaps not surprising that the genes encoding many centrosomal proteins were found in genetic screens for lethality or sterility aimed at identifying cell cycle mutants. One particular class of *maternal-effect-lethal* (MEL) mutants identify maternally-expressed genes, required for the first 2 hr of syncytial embryo development when nuclei follow rapid cycles of alternating S- and M-phase, first at 10-min intervals and then gradually lengthening to ∼20 min, to allow some zygotic transcription from cycle 10 onward. This massive increase in nuclei during syncytial development requires a dowry of maternally-provided cell cycle regulatory molecules. A mother homozygous for mutations in such genes reaches adulthood because her own heterozygous mother (the grandmother) has provided the wild-type gene product, but she is unable to contribute any wild-type protein to her own offspring.

The centrosomes play an important role in organizing MTs through these rapid cycles of nuclear division. The first rounds of nuclear division occur within the interior of the embryo, but later nuclei and centrosomes migrate together to the cortex of the syncytium. Here, the centrosomes induce dramatic reorganization of the cortical actin, which forms an apical cap above each nucleus and its apically-positioned centrosome. Repeated rounds of DNA replication in embryos derived from mothers carrying the MEL mutation *giant nuclei* (*gnu*), leads to the dissociation of centrosomes from nuclei (Freeman *et al.* 1986; Freeman and Glover 1987; Lee *et al.* 2003; Renault *et al.* 2003). Centrosomes can also be uncoupled from nuclei by inhibiting DNA replication (Raff and Glover 1988). Such independently duplicating centrosomes migrate to the cortex of the embryo at the blastoderm stage. Remarkably, when the first centrosomes reach the posterior pole, they encounter the maternally-deposited polar granules and this interaction is sufficient to trigger the formation of the first cells, the primordial germ cells, and in this case without the participation of nuclei (Raff and Glover 1989).

Many of the structural components of the mitotic apparatus, including centrosomal molecules, are stable and their maternal dowry can perdure until the late larval stages. This is helped by the fact that throughout larval development there is very little mitotic cell division, and tissues become bigger largely through the endo-reduplication of their genomes. However, the imaginal tissues and the Central Nervous System (CNS) do have to proliferate to form the adult structures and failure to do so leads to lethality at this stage. This phenotype, first recognized by Gatti and Baker, has been used successfully to identify many cell cycle proteins (Gatti and Baker 1989). Some genes encoding cell cycle proteins can have one set of mutant alleles that exhibit maternal-effect-lethality and another late larval lethal set. The original mutant alleles of the centrosomal regulators *polo* and *aurora*, for example, were first isolated as weak hypomorphic maternal effect mutants from Christiani Nüsslein-Volhard’s collection (Sunkel and Glover 1988; Glover 1989; Leibowitz 1990). Later, stronger hypomorphic alleles for both *polo* and *aurora* were identified showing late larval/pupal lethality (Glover 1989; Llamazares *et al.* 1991; Glover *et al.* 1995).

Polo encodes a protein kinase with periodic activity in the syncytial nuclear division cycles (Llamazares *et al.* 1991; Fenton and Glover 1993), which functions at multiple stages of the cell cycle. Its subcellular activity can be tracked using its localization; it is on the centrosomes until anaphase and associated with the central spindle and midbody during cytokinesis [reviewed by Archambault and Glover (2009)]. Aurora has similarly diverse functions mediated by two forms of the kinase: Aurora A (encoded by the original *aurora* gene) whose roles are largely centrosomal and Aurora B, the catalytic partner of the inner centromere protein INCENP, part of the chromosome passenger complex (Giet and Glover 2001; Giet *et al.* 2002).

### Screens in cell culture

While the identification of mutants was the great driving force in initiating the building of the “parts catalogue,” the fact that many of the core functions of centrosomes are common features of all dividing cells led to the advantageous application of RNA interference (RNAi) as a major route to gene discovery. Systematic RNAi-mediated knockdown of all protein kinases in the fly genome led to the identification of their cell cycle roles and the discovery of Polo-like kinase 4 (plk4) as the key regulator of centriole duplication (Bettencourt-Dias *et al.* 2004). A genome-wide screen carried out by Goshima and Vale then identified proteins required for the functional integrity of the mitotic apparatus (Goshima *et al.* 2007), including the key genes required for the centriole duplication cycle with the *anastral spindle* (*ana*) phenotype (see discussion of *Centriole duplication* below). Finally, in a focused genome-wide screen, Dobbelaere *et al.* (2008) identified the majority of genes required for centriole duplication and maturation of centrosomes, confirming the function of previously known gene products as well as identifying some new players.

## Centrosome Architecture: Putting the Parts Together

One of the main advantages of *Drosophila* as model organism is the possibility of studying different kind of cell division in different tissues at different developmental stages. It is well-established that different cell lineages and developmental stages show different centriole architecture and centrosome organization (González *et al.* 1998). EM was, at first, the only way of following the behavior of the *Drosophila* centriole. Embryonic and cultured cell centrioles are composed of doublets of MTs which are ∼0.2 µm long and 0.2 µm wide (Moritz *et al.* 1995; Debec *et al.* 1999), and apparently uniform in structure; they have a “cartwheel” structure consisting of a central hub and nine radially-arranged spokes along their entire length (Callaini *et al.* 1997; Debec *et al.* 1999). Centrioles from differentiated tissues such as wing cells and interommatidia sensory bristles display triplets of MTs and have no cartwheel (Debec *et al.* 1999). Unlike mammalian cells, there are no visible structures associated with the external MT wall called distal or subdistal appendages (Callaini *et al.* 1997), and mother and daughter centrioles can only be distinguished because the daughter lies orthogonal to the proximal end of the mother (Rieder and Borisy 1982). However, in male germ stem cells (mGSCs), the mother centriole has triplets of MTs, whereas daughters show a mixture of doublets and triplets (Gottardo *et al.* 2015b). The centrioles from spermatocytes are very much longer than those at other developmental stages, as we will discuss below.

**Figure 2:**
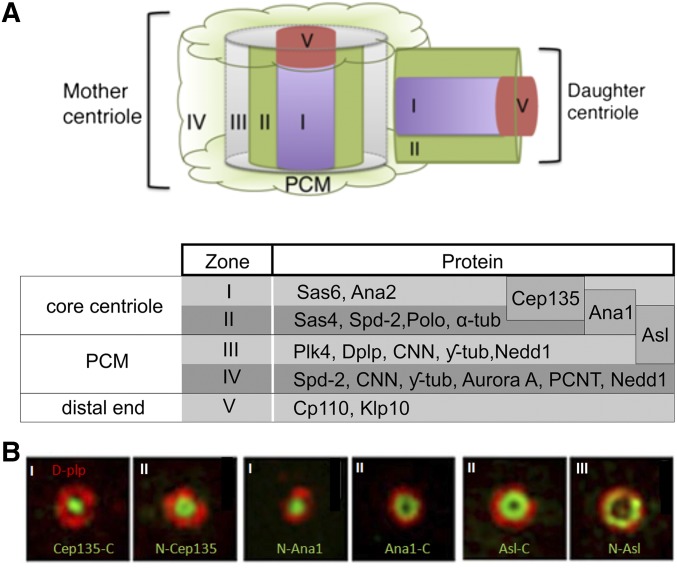
The zonal organization of the centrosome. (A) Zones of *Drosophila* centrosome as defined by superresolution microscopy (Fu and Glover 2012). This recognized a core components present in Zones I and II in both the mother and the procentriole (the daughter) at mitotic entry. Zone III is only present on the mother centriole surrounded by PCM (Zone IV) at mitotic entry. Zone II is completed around the daughter during passage through mitosis. Molecular components of the different zones are tabulated. (B) Supperresolution images showing localization of the N- and C-termini (green) of the molecular network that extends from Zone I to Zone III of the centriole, which is built during the process of centriole-to-centrosome conversion (see text and Fu *et al.* 2016). DPlp (red) serves as a reference point for Zone III.

In recent years, superresolution light microscopy has been invaluable in positioning the multiple component parts of centrioles and their surrounding PCM and has defined distinct zones of the *Drosophila* centrosome (Figure 2) (Fu and Glover 2012; Mennella *et al.* 2012; Dzhindzhev *et al.* 2014; Fu *et al.* 2016). Genetic screens in *Caenorhabditis elegans* first identified Sas5 and Sas-6 as proteins that initiate centriole duplication [reviewed by Strnad and Gönczy (2008)]. Their respective fly counterparts, Ana2 and Sas-6, lie at the very core (Zone I) of the centriole. Sas-6 is a conserved coiled-coil protein that has N-terminal globular domains that dimerize to form the unit of the ninefold symmetrical cartwheel (Kitagawa *et al.* 2011; Van Breugel *et al.* 2011; Cottee *et al.* 2015). Sas-6 interacts not only with Ana2 but also with Cep135, the counterpart of the *Chlamydomonas bld10* gene, whose bald mutant phenotype reflects loss of flagellae (Matsuura *et al.* 2004). Cep135 has a radial orientation with its C-terminal part in Zone I and N-terminal part projecting outward, and it is required for a daughter to be able to become a mother (see below) (Fu *et al.* 2016).

The main component of Zone II is Sas-4, which provides a connection between the cartwheel and the MT wall and which physically interacts both with α/β tubulin dimers and Sas5/Ana2 (STIL in human cells) (Hsu *et al.* 2008; Tang *et al.* 2011). The ring of Sas-4 in cultured DMel cells is ∼200 nm diameter, similar to the centriole diameter seen by EM (Fu and Glover 2012). This is in accord with immuno-EM studies that locate Sas-4 at both the internal and external surfaces of the centriole wall and in the PCM (Gopalakrishnan *et al.* 2011). Polo kinase and Spd-2, usually thought of as components of the PCM that surround the mother centriole in mitosis (see below), are also present in Zone II during interphase.

Zone III marks the foundation upon which the PCM will be laid in mitosis. The coiled-coil protein DPlp is frequently used as a marker for Zone III. Very little is yet known about the function of this protein, which is arranged radially around the centriole wall with the C-terminal part forming a ring of ∼86 nm of radius and the N-terminal part radiating outward within the PCM at ∼138 nm (Mennella *et al.* 2012). Moreover, DPlp localization seems to differ in cultured cells and *Drosophila* embryos where it also colocalizes with Centrosomin (Cnn) in the PCM to form MT-dependent extrusions called “flares” (reviewed in Conduit *et al.* (2015b)). Asl and Plk4 are also present in Zone III where their distribution overlaps with DPlp. This organization is largely similar in *Drosophila* spermatocyte centrioles where Asl extends along the entire centriole and DPlp is restricted to the proximal part (Fu and Glover 2012).

The majority of the PCM components, including Cnn, DSpd-2 and γ-tubulin, are recruited in mitosis to form Zone IV.

The final region, Zone V, is occupied by CP110 and its partner proteins. This complex forms a cap or plug at the distal end of mother and daughter centrioles and is involved in centriole length regulation (Schmidt *et al.* 2009; Fu and Glover 2012). In primary spermatocyte centrioles, CP110 is present in young centrioles but its levels diminish during centriole elongation and it is completely absent from the giant centrioles associated with a primary cilium (Fu and Glover 2012) (see below).

Superresolution microscopy provided information about protein localization and gave us clues about protein–protein interactions (PPIs). However, further molecular studies were needed to understand when and where PPIs happen and to identify transient interactions. To this end, Galletta and collaborators generated a centrosome interactome using a yeast two-hybrid (Y2H) screen to identify the PPIs among 21 centrosome proteins, and then used this information to gain some understanding of protein organization within the centrosome *in vivo* (Galletta *et al.* 2016b).

## Centriole Duplication

**Figure 3:**
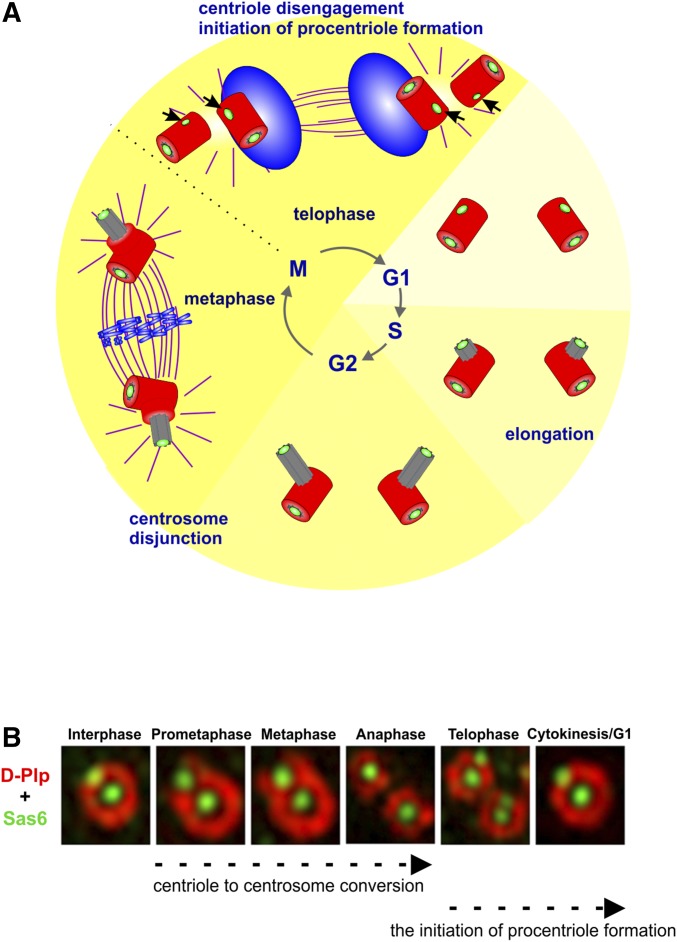
Key steps of the centriole duplication cycle. (A) Schematic representation of the initiation of procentriole formation (black arrows), centriole elongation, and centriole-to-centrosome conversion. Green, cartwheel; gray, centriole; red, pericentriolar material; blue, chromosomes; violet, microtubules. See text for details. (B) Superresolution images depicting the extension of Zone III (DPlp staining in red) around the daughter centriole in passage through mitosis in the process of centriole-to-centrosome conversion, and the initiation of procentriole formation with the recruitment of Sas-6 (green) onto both mother and daughter centrioles as they disengage at telophase.

Centriole duplication is generally described as beginning in G1 as this is the stage at which centriolar MTs become apparent using EM. However, using superresolution microscopy in cultured *Drosophila* cells, it has become evident that, in this organism, the initiation of procentriole formation occurs prior to G1, immediately following the disengagement of mother and daughter centrioles at the end of mitosis in telophase (Dzhindzhev *et al.* 2014). This occurs with the recruitment of the key cartwheel components, Sas-6 and Ana2, and the procentriole will only be finally completed by the time of disengagement once the cell passes through the subsequent mitosis. Following cytokinesis, the newborn cells each have two loosely connected centrioles. New procentrioles begin to grow in length perpendicular to these inherited centrioles (the old and new mothers) during G1 and G2 until they reach their mature length. In preparation for mitosis, the mother centrioles begin to accumulate PCM and so nucleate increasing numbers of MTs for spindle assembly. In mammalian cells, the loose fibrous tethers between the mother and its old daughter then resolve, permitting centrosomes to disjoin and separate to opposite sides of the nucleus, where they organize opposing poles of the mitotic spindle. During mitosis, the procentriole (the new daughter) becomes competent to replicate and recruit PCM, in a process known as centriole-to-centrosome conversion (see below). Mother and daughter pairs will disengage at the end of mitosis. Thus, after cell division, each daughter cell inherits a mother and a newly completed daughter centriole, each of which have initiated formation of the cartwheel of the new procentriole by recruiting Ana2 and Sas-6, and the centriole cycle begins once more (Figure 3).

The regulatory protein kinases that lie at the head of the centriole duplication cascade, ZYG1 in the worm and Plk4 in the fly, are sufficiently different in sequence that it was some years before it was realized that they were true orthologs. Loss of *Drosophila* Plk4 function as a result of mutation or RNAi leads to centrosome loss, a disorganized mitotic spindle, and an inability to form basal bodies (Bettencourt-Dias *et al.* 2005). Conversely, overexpression of Plk4 results in centrosome amplification in embryos and in spermatocytes (Rodrigues-Martins *et al.* 2007b; Lopes *et al.* 2015). Centrosome amplification also occurs in unfertilized eggs that overexpress Plk4 and because, as we saw earlier, centrioles are eliminated from the female germline during oogenesis and because there is no incoming sperm to provide a basal body, this indicates Plk4’s ability to drive centriole formation *de novo* as well as promote canonical duplication (Rodrigues-Martins *et al.* 2007b).

Since the triggering of centriole duplication depends upon and is sensitive to Plk4 levels, the amounts of Plk4 must be tightly spatiotemporally regulated to ensure that the centriole duplication occurs once per cell cycle. The SCF/Slimb ubiquitin protein ligase complex limits the amount of Plk4 during the cell cycle by catalyzing Plk4 ubiquitylation, thus, permitting degradation by the proteasome (Cunha-Ferreira *et al.* 2009; Rogers *et al.* 2009). The signal for SCF/Slimb-mediated ubiquitylation is the auto-phosphorylation of Plk4; thus, Plk4 is a suicide kinase that is degraded once its level reaches a threshold (Cunha-Ferreira *et al.* 2013). Another layer of Plk4 regulation may be given by the PP2A/Twins phosphatase, which was suggested to counteract Slimb-mediated degradation of Plk4 in mitosis and thus to help stabilize Plk4 in this cell cycle stage (Brownlee *et al.* 2011).

At the onset of centriole duplication, Plk4 recruitment and stabilization on the *Drosophila* centriole is dependent upon Asl, a product of the *asterless* gene and ortholog of the mammalian protein Cep152. The N-terminal domain of Asl binds to the cryptic Polo box domain (aka Polo boxes 1 and 2) of Plk4, an interaction that is indispensable for centriolar localization of Plk4 in *Drosophila* (Dzhindzhev *et al.* 2010). Mutant forms of Asl that are unable to bind Plk4 act as dominant inhibitors of centriole duplication when overexpressed in cultured cells. Just as with its kinase partner, elevating the level of Asl results in centrosome amplification in both *Drosophila* embryos and cell lines (Dzhindzhev *et al.* 2010).

Asl not only targets Plk4 to centrioles but also modulates Plk4 stability and activity. In interphase, the Asl N-terminus promotes Plk4 homodimerization and autophosphorylation, whereas the Asl C-terminus stabilizes Plk4 during mitosis, contributing to the ability of overexpressed Asl to drive centriole amplification (Klebba *et al.* 2015).

Thus, procentriole formation can be triggered at multiple sites when Plk4 is overexpressed (Bettencourt-Dias *et al.* 2005; Rodrigues-Martins *et al.* 2007b), when its SCF-dependent proteolysis is prevented (Cunha-Ferreira *et al.* 2009; Rogers *et al.* 2009), and when expression of its targeting subunit is elevated (Dzhindzhev *et al.* 2010).

In the Y2H screen referred to above, it was shown that Plk4 directly binds Ana1, Ana2, Asl, CP110, Cep135, and Plk4 itself. The binding to Cep135 is mediated by the Polo Boxes 1–2 cassette of Plk4, then Cep135 is phosphorylated by Plk4 and this phosphorylation is critical for positioning Asl on centrioles in *Drosophila* spermatocyte giant centrioles (Galletta *et al.* 2016b).

Once recruited to the centriole, Plk4 phosphorylates the core centriole component, Ana2. Once phosphorylated on its STAN motif, Ana2 is able to bind to and recruit the essential cartwheel component, Sas-6. A mutant form of Ana2 that cannot be phosphorylated on this site is still able to localize to the centriole but does not recruit Sas-6 (Dzhindzhev *et al.* 2014). The four residues phosphorylated by Plk4 are highly conserved and phosphorylation of the human Ana2 counterpart, STIL, at these same sites by Plk4 is required for STIL to bind to human Sas-6 (Ohta *et al.* 2014).

A major objective of the initiation of centriole duplication is the recruitment of Sas-6 to the site on the centriole at which the procentriole will assemble. Sas-6 is essential for correct cartwheel and thus centriole structure, and its mutants in *Drosophila* show deficiencies in the ninefold symmetry of the centriole (Rodrigues-Martins *et al.* 2007a). As in many other species, *Drosophila* Sas-6 proteins form dimers through their C-terminal coiled-coil domain (Kitagawa *et al.* 2011; Van Breugel *et al.* 2011, 2014; Hilbert *et al.* 2013). Sas-6 dimers further homooligomerize through an N-terminal headgroup interaction and form a ring structure from which coiled-coil domains emanate as spokes, resembling the shape of a cartwheel with ninefold symmetry as it is visualized by EM. Unlike the case of human cartwheel, where Sas-6 and STIL dissociate from the centriole and are degraded from the onset of anaphase until late G1 phase (Strnad *et al.* 2007; Arquint and Nigg 2014), *Drosophila* Sas-6 is stable at the core of the centriole once it is incorporated and can be observed both on the mother and daughter centriole from the point of the initiation of procentriole formation and throughout the cycle (Dzhindzhev *et al.* 2014).

In contrast to Plk4 or Asl, overexpression of Sas-6 in eggs or embryos does not lead to proper centriole formation but rather to amorphous tube-like aggregates (Rodrigues-Martins *et al.* 2007a). However, if Sas-6, Ana2, and Plk4 are overexpressed in spermatocytes they appear to lead to centriole overduplication (Stevens *et al.* 2010b). These phenotypes accord with the model that Sas-6 recruitment is dependent on Plk4-mediated Ana2 phosphorylation.

Both Ana2 and Sas-6 have conserved coiled-coil domains and form homooligomers to perform their centriolar functions. The central coiled-coil domain of Ana2 forms a homotetramer and mutations perturbing tetramerization perturb centriole duplication in dividing larval neuroblasts (NBs), suggesting the importance of the oligomerization of this centriolar component in procentriole formation and Sas-6 recruitment. Sas-6 proteins are recruited to the centrioles as homodimers and mutations preventing Sas-6 homooligomerization also perturb centriole duplication in the rapidly dividing embryonic nuclei, but not in slower dividing larval NBs and spermatocytes (Cottee *et al.* 2015).

## Centriole Elongation

The regulation of centriole length may depend upon multiple proteins. In mammalian cells, it is shown that overexpression of either SAS-4/CPAP (centrosomal P4.1-associated protein) or its interacting protein CEP120 results in excessively long centrioles. Conversely, loss of these proteins inhibited CPAP-induced centriole elongation (Comartin *et al.* 2013; Lin *et al.* 2013). The elongation of centrioles brought about by overexpression of CPAP can be counteracted by CP110 (Kohlmaier *et al.* 2009; Schmidt *et al.* 2009; Tang *et al.* 2009), which forms a plug-like structure at the distal end of the centriole; depletion of CP110 in cultured human cells leads to abnormally long centrioles (Schmidt *et al.* 2009). These events are not so clear-cut in *Drosophila*. What is evident is that the length of the centriole must be regulated through the balance of the polymerization and depolymerization of the centriolar MTs. One important regulator of centriole length in *Drosophila* is Klp10A, a kinesin-like protein that depolymerizes MTs (Rogers *et al.* 2004; Delgehyr *et al.* 2012). In absence of Klp10A, both *Drosophila* cultured cells and spermatocytes develop longer centrioles that show incomplete ninefold symmetry at their ends and tend to undergo fragmentation (Delgehyr *et al.* 2012). Klp10A interacts with the centriole capping protein CP110 (Delgehyr *et al.* 2012). However, in contrast to the centriole elongation seen following CP110 depletion in mammalian cells (Schmidt *et al.* 2009), CP110 downregulation leads to shortening in centriole length in cultured *Drosophila* cells (Delgehyr *et al.* 2012). Such shortening can be overcome by codepletion of CP110 with Klp10 resulting in longer centrioles than usual (Delgehyr *et al.* 2012). In contrast, Franz *et al.* (2013) reported moderate centriole elongation in larval wing discs and NBs in the absence of CP110. Conversely, overexpression of CP110 gave an ∼20% reduction in the length of wing disc centrioles. However, the elongation observed did not affect the whole centriole structure and was considered to be due to MT protrusions emanating from the distal ends of centrioles (Franz *et al.* 2013). The tissue-specific consequences of CP110 depletion are reflected in the lack of any effect upon the size and structure of centrioles/basal bodies in spermatocytes. Many aspects of CP110 function demand further study. It is reported, for example, that overexpression of several centriole components in a CP110 mutant background results in elongation of centrioles, although this cannot be observed in presence of CP110. Interestingly, CP110 overexpression can also suppress centriole amplification induced by an excess of Plk4 in larval NBs through unknown mechanisms (Franz *et al.* 2013). It is also reported that CP110 function is downregulated by Neurl4 protein, which concentrates at the centrosomes and may have a regulatory role in primordial germ cell formation (Jones and Macdonald 2015). Although, the precise mechanism of CP110 downregulation by Neurl4 is not well-established, studies on human Neurl4 suggest that this protein promotes CP110 ubiquitylation (Li *et al.* 2012).

Other factors important for centriole elongation or for the maintenance of their length, at least in spermatocytes, are Cep135/Bld10 (Mottier-Pavie and Megraw 2009), Proteome Of Centriole 1 (Poc1) (Blachon *et al.* 2009), and Bug22 (Mendes Maia *et al.* 2014). Spermatocyte centrioles and basal bodies are shorter than wild-type in *bld10* and *poc1* mutants, while in *bug22* they are longer than their wild-type counterparts and assemble centriole pairs with strange bends and/or arrangements.

It was recently reported that *asl* mutant centrioles are longer than their wild-type counterparts and that they display proximal centriole proteins along the entire length of the centriole. In this case, the CP110 partner Cep97 acts downstream of Asl in centriole length control and appears to be acting independently of CP110 (Galletta *et al.* 2016a).

Ana1 is also involved in the process of centriole elongation, which it promotes in a dose-dependent manner in spermatocytes; centrioles are slightly longer when Ana1 is overexpressed, and slightly shorter when *ana1* gene dosage is halved. This function depends on the Ana1 N-terminal region (Saurya *et al.* 2016), which was reported to interact with Cep135 in the process of centriole-to-centrosome conversion in cultured cells (Fu *et al.* 2016). Additionally, Cep135, Ana1, and Asl also affect centriole elongation *in vivo* evoking the question of whether or not the two pathways may be interconnected. Further studies will be needed to define the relationships between these elements that regulate centriole length.

## Centriole-to-Centrosome Conversion

Although the newly formed centrioles reach their full length in early mitosis, they have no inherent ability to duplicate or to organize the PCM. The capacity of duplication and PCM recruitment are acquired after passage through mitosis as a result of Plk1-dependent modification, first demonstrated in cultured human cell lines (Wang *et al.* 2011). The process of centriole-to-centrosome conversion (Figure 1 and Figure 3) requires the presence of specific conserved scaffolding proteins (Izquierdo *et al.* 2014; Fu *et al.* 2016). Fu *et al.* (2016) identified a conserved architectural network of Cep135-Ana1-Asl that is key for centriole-to-centriole conversion in both *Drosophila* and human cells. The C-terminal part of Cep135 lies within Zone 1 and the molecule extends outward with its N-terminus lying in Zone II. Cep135 can interact with itself at both termini to form an extended dimer or multimer (Galletta *et al.* 2016b). This N-terminal part of Cep135 physically interacts with the N-terminal part of Ana1, which has its N-terminus in the outer part of Zone I and its C-terminal part in the outer part of Zone II. The C-terminal part of Ana1 interacts with the C-terminal part of Asl, which extends to have its N-terminus in Zone III. The components of this network are loaded sequentially during mitotic progression starting from inner to outer centriole. Depletion of Ana1 affects the recruitment of Cep135, suggesting a Cep135-Ana1 interaction in the first stage of centriole-to-centrosome conversion. The outermost component of the network Asl is recruited by Ana1. Thus, Ana1 forms a molecular structure that connects proteins of the inner centriole to the outermost proteins. A similar network of orthologous proteins is found in human cells and their loading is necessary before the centriole is able either to duplicate or recruit PCM (Fu *et al.* 2016).

Cep135 can also directly interact with Asl and superresolution measurements suggest that that the termini of these proteins may overlap. The Cep135 N-terminal part is a substrate of Plk4 and phosphorylation relieves autoinhibitory effect in full-length Cep135, thereby allowing it to interact with the Asl C-terminal (Galletta *et al.* 2016b). Further investigation is necessary to determine if Ana1’s bridging property activity is required in all cell types or if, in some circumstances, Ana1 and Cep135 could have partially redundant functions.

Centriole disengagement has been described as a licensing step for centriole duplication from experiments carried out in vertebrate systems (Tsou and Stearns 2006). Although the precise mechanisms of centriole disengagement are not clearly known in *Drosophila*, it has been suggested that recruitment of Asl onto the daughter centriole occurs only once mother and daughter have separated at the end of mitosis and that this is the event that provides the duplication license (Novak *et al.* 2014). However, in the conventional cycles of cultured cells, the very first event in duplication can be followed by incorporation of Ana2 and Sas-6 onto both the mother and the daughter’s procentrioles immediately after centriole disengagement in telophase (Dzhindzhev *et al.* 2014). Disengagement in human cells requires the activity of Separase and the Polo kinase in late mitosis (Tsou *et al.* 2009). An involvement of Polo in disengagement in *Drosophila*, in line with findings in human cells, is provided by the observation that pharmacological inhibition of Polo kinase in testis prevents centriole separation that usually occurs during the anaphase of the first meiosis (Riparbelli *et al.* 2014).

The precise roles of Polo and Separase remain uncertain. A classical substrate of Separase is a component of the cohesion complex, best known for its requirement to maintain sister chromatid cohesion. Centriole disengagement has also been proposed to be dependent upon Separase’s cleavage of Cohesin in mammals but such an involvement in *Drosophila* has been challenged by experiments carried out in embryos (Oliveira and Nasmyth 2013). These authors showed that artificial cleavage of the cohesin subunit, Scc1, in metaphase-arrested embryos did not lead to centriole disengagement. The possibility that other substrates of Separase are important for centriole disengagement is supported by the finding that, in mammals, pericentrin B is specifically cleaved at the exit of mitosis (Lee and Rhee 2012; Matsuo *et al.* 2012). Further studies are necessary to investigate if such a mechanism is active also in *Drosophila*.

Centriole disengagement does not occur until after exit from the mitotic state. Accordingly, disengagement can be promoted in metaphase-arrested embryos by treating them with the mitotic Cdk1 inhibitor p21 (Oliveira and Nasmyth 2013). Thus, it would seem unlikely that binding of Polo to a docking site created through Cdk1-mediated phosphorylation would have importance for centriole disengagement. Polo has been shown to be recruited to Sas4 phosphorylated at Thr200 by Cdk1/Cyclin B (Novak *et al.* 2016) and indeed, disrupting this recruitment of Polo to daughter centrioles does not appear to alter the efficiency of centriole disengagement. Thus, it would seem that the pool of Polo on the mother centriole or in the cytoplasm is sufficient to drive this event (Novak *et al.* 2016). Further investigations are necessary to understand the exact roles of Separase and Polo in centriole disengagement.

## Centrosome Maturation: Mitotic Recruitment of PCM

It was Boveri in the 1890s who first described how the centrosome became enlarged in mitosis. Such centrosome maturation is a result of the expansion of the PCM, the cytoplasmic MT-nucleating material that surrounds the core centrioles (Figure 1). The MT-nucleating component of the PCM is γ-tubulin, which exists as a dimer in a complex with two other molecules that are the counterparts of Spc98 and Spc97 of budding yeast. These tetrameric complexes form part of a larger ring complex (γ-TuRC) that forms the foundation for building the MT. Mutants of γ-tubulin in *Drosophila* disrupt the structure of MTOCs during mitosis (Sunkel *et al.* 1995) as do mutants of the other subunits, for example the late larval lethal mutant *discs degenerate-4* (*dd4*), which encodes the Spc98 counterpart (Barbosa *et al.* 2000).

It has long been clear that Polo kinase plays an essential role in recruiting PCM during centrosome maturation. This was first evident in the original *polo* mutant that had defective spindle poles and an abnormal distribution of the centrosomal protein CP190 (Sunkel and Glover 1988), and in the stronger hypomorphic alleles studied by Donaldson and collaborators (Donaldson *et al.* 2001) in which γ-tubulin cannot be detected at spindle poles. These findings were echoed following the development of pharmacological inhibitors of Polo that prevent nucleation of MTs by partially purified preparations of *Drosophila* centrosomes (McInnes *et al.* 2006). The ability to mobilize elevated levels of Polo kinase at centrosomes in mitosis is in part to be achieved through the Hsp90 chaperone that appears to enable Polo’s stability. *Hsp90* mutants display defects in centrosome maturation, as do cells treated with the Hsp90 inhibitor geldanamycin. Cytoplasmic extracts treated with geldanamycin can no longer rescue the ability of salt-stripped preparations of partially purified centrosomes to nucleate MTs, a defect that can be rescued by adding back more active Polo kinase. This highlights the importance of chaperones to protein kinases that are required to function in these periods with intense activity to rapidly organize cytoskeletal elements (de Cárcer *et al.* 2001).

Before PCM can be recruited, a necessary foundation must be created. This foundation is present around the mother centriole but is only built upon the daughter as she passes through mitosis in the process of centriole-to-centrosome conversion (see above). One component of this foundation is the conserved pericentrin-like protein (DPlp), which tightly associates with the wall of mother and mature daughter centrioles (Fu and Glover 2012; Mennella *et al.* 2012). Flies lacking DPlp show defects in PCM assembly in mitosis, although by no means as severe as those seen in the absence of either Cnn or Spd-2 (Martinez-Campos *et al.* 2004; Lerit *et al.* 2015; Richens *et al.* 2015), two of the principal PCM components that are recruited through the activity of Polo kinase and are in turn required for the recruitment of the γ-tubulin ring complex (Figure 1).

The gene encoding Cnn, originally discovered by Kaufman’s lab (Heuer *et al.* 1995), encodes two isoforms, each with a number of splice variants. The long isoform (Cnn-LF) is a major PCM component that exhibits dynamic flaring on the centrosomes of syncytial embryos throughout the nuclear division cycle (Megraw *et al.* 2002; Conduit *et al.* 2010). A striking feature of *cnn* mutants is that, although they fail to form functional PCM during mitosis, they still are able to segregate chromosomes. In fact, they are able to support development but give rise to sterile adults (Megraw *et al.* 2001). A similar phenotype was later found by Basto *et al.* (2006) in Sas-4 mutants that lack centrioles and so fail to form any cilia or flagellae. However, centrioles and the centrosomes that they organize are required for the syncytial nuclear division cycles and for male fertility (Rodrigues-Martins *et al.* 2008). Moreover, a recent study of acentrosomal cells in the wing disc has revealed prolonged spindle assembly, chromosome missegregation, DNA damage, misoriented divisions, and apoptosis, indicating the importance of centrosomes for cell division in fly epithelia (Poulton *et al.* 2014).

Chemical inhibition of polo kinase in cultured *Drosophila* cells blocks the localization of Cnn to the centrosomes at mitotic onset (Fu and Glover 2012). However, there may be some variation between cell types or between the behavior of different isoforms, as in another study some Cnn remained at the spindle poles in larval NBs of strong hypomorphic *polo* mutants (Donaldson *et al.* 2001). The phosphorylation of Cnn by Polo is complex. Conduit *et al.* (2014a) have described a small central region of Cnn, which they term the phosphoregulated multimerization domain (PReM), that has numerous phosphorylation sites, some of which can be phosphorylated by Polo kinase. Its phosphomimetic mutants form oligomeric structures *in vitro* and *in vivo*, whereas mutant forms that cannot be phosphorylated are recruited to the PCM but cannot support PCM expansion. The Kaufman group identified two closely spaced amino acid residues present in an N-terminal part of a subset of Cnn isoforms, but the phenotypic consequences of mutating these sites is complex (Eisman *et al.* 2015, 2016).

The MT-nucleating properties of Cnn are also required in the acentriolar divisions of female meiosis to make the characteristic central aster of MTs that forms between the tandem spindles of the second meiotic division (Riparbelli and Callaini 2005). Although it is unclear why loss of the short isoform should allow all four products of the meiotic divisions to undergo nuclear division cycles (Eisman *et al.* 2015), this is a similar phenotype to that of embryos derived from *polo^1^*-mutant mothers, pointing to a shared role for Polo kinase and Cnn-SF in this process. Given the complexity of Cnn isoforms and of their modification by phosphorylation, it will be some time before the multiple functions of Cnn are completely understood.

Other than Polo kinase and Cnn, one other molecule, Spd-2, plays a key role in recruiting the γ-tubulin ring complex. This contrasts with its role in *C. elegans*, where Spd-2 was first described as being required for recruitment of the *ZYG1* polo-like kinase that drives centriole duplication in this organism. The main role of Spd-2 in *Drosophila* was first apparent from the diminished recruitment of PCM in *spd-2* mutant somatic cells and spermatocytes (Dix and Raff 2007; Giansanti *et al.* 2008). Fu and Glover (2012) showed that the amounts of Cnn, γ-tubulin, Spd-2, and Polo all expand upon mitotic entry specifically in Zone IV around the mother centriole, the daughter being incompetent to do so until it has progressed through the process of centriole-to-centrosome conversion. Depletion of either Cnn or Spd, or inhibition of Polo kinase with BI2536 treatment, prevented the accumulation of γ-tubulin around the centriole, with Cnn depletion being the most effective. Inhibition of Polo or depletion of Cnn were equally efficient at preventing the recruitment of Spd-2. Finally, whereas Polo inhibition prevented Cnn recruitment, Spd-2 depletion only reduced final Cnn levels by about one-half. Thus, in this cell type, γ-tubulin recruitment appears primarily dependent upon Cnn that in turn is absolutely dependent upon Polo kinase. Spd-2 appears to facilitate this process and its recruitment into the PCM requires both Cnn and Polo (Fu and Glover 2012). Using a very different technical approach, injecting neutralizing antibodies into the syncytial embryo, Conduit *et al.* (2014b) reached similar conclusions. They showed that, in the absence of either Spd-2 or Cnn, PCM assembly was greatly reduced, whereas if both Spd-2 and Cnn were absent the PCM was not assembled. The findings were consistent with a role for Spd-2 facilitating Cnn recruitment and Cnn in turn helping to maintain Spd-2, thus setting up a positive feedback loop that drives centrosome maturation (Figure 1) (Conduit *et al.* 2014b). The PCM appears to be a dynamic lattice. Both Cnn and Spd-2 are recruited to the centriole and spread outward in constant flux through the rest of the PCM (Conduit *et al.* 2010, 2014b; Lerit *et al.* 2015).

This model for PCM recruitment is very much in line with findings from an *in vitro* system developed to study centrosome maturation in *C. elegan*s. Here, the coiled-coil protein SPD5 carries out equivalent function to Cnn, and its ability to be incorporated into networks is accelerated by Plk1 and Spd-2. The resulting SPD5 network serves as the assembly platform for all other PCM proteins (Woodruff *et al.* 2015). Together, these studies identify a conserved core system for centrosome maturation through PCM recruitment and, although the details may vary between organisms, the underlying principles are now defined.

What is less well-characterized is the foundation of the centriole upon which this mitotic platform is built. It has been proposed in *Drosophila* that the centriolar protein Sas-4 can provide such a foundation. However, the role of Sas-4 in PCM recruitment has been a little controversial. Sas-4 functionally interacts with Asl and, if a mutant form of Asl unable to bind Plk4 is expressed in syncytial embryos, it drives the formation of MTOCs that lack centrioles at their core (Dzhindzhev *et al.* 2010). This phenocopies the phenotype resulting from overexpression of Sas-4. Thus, it would seem that the Sas-4-Asl complex may be able to organize PCM and so nucleate MTs. This would be consistent with a report that Sas-4 is able to scaffold so-called cytoplasmic S-CAP complexes by binding to Asl, Cnn, DPlp, and CP190 via its N-terminal domain, and that Sas-4 can tether such complexes to the centrosome via its C-terminal domain (Gopalakrishnan *et al.* 2011). These workers have gone on to suggest that tubulin-GTP prevents Sas-4 from forming protein complexes for PCM recruitment and that tubulin-GDP promotes this. However, Conduit *et al.* (2015a) have reported that Cnn, Spd-2, and Asl are not recruited to the mother centriole as part of a complex with Sas-4 and, thus, PCM recruitment does not appear to require cytosolic S-CAP complexes in fly embryos.

## Connecting Centrosomes and Spindle Poles

As the PCM must make functional contacts with MT minus ends, it becomes difficult to define whether a given minus end-associated MAP is a PCM component or not. One case in point is Asp, product of the *abnormal spindle* gene, and one of the first identified substrates of *Drosophila* Polo kinase (Tavares *et al.* 1996; Saunders *et al.* 1997; do Carmo Avides and Glover 1999, 2001). Asp protein is enriched at the spindle poles that typically become widely splayed in *asp* mutants (do Carmo Avides and Glover 1999). Partially purified *Drosophila* centrosomes are dependent upon Asp to nucleate rhodamine-labeled tubulin on microscope slides. Treatment of such centrosome preparations with high salt strips away the PCM and they lose MT-nucleating ability. Restoration of nucleating activity by a high-speed supernatant from a cytoplasmic extract is ineffective if it has been depleted of either the ƴ-tubulin ring complex (Moritz *et al.* 1998) or Asp, either by mutation or immunodepletion (do Carmo Avides and Glover 1999). Adding back purified Asp protein to the Asp-deficient extract will rescue its ability to nucleate MTs, although it has to be phosphorylated by Polo kinase to be active. Cytoplasmic extracts of *polo* mutant embryos are unable to restore MT-nucleating activity to salt-stripped centrosomes but can do so if they are supplemented with phosphorylated Asp protein (do Carmo Avides *et al.* 2001). A recent study showed that Asp can focus the minus ends of MTs independently of Ncd and that this can occur at points distant from the poles to give focused MT clusters that can migrate to the poles (Ito and Goshima 2015). Asp has long been known to harbor a calmodulin-binding motif (Saunders *et al.* 1997); deletion of this motif renders the protein unable to cross-link and focus MTs and results in the loss of centrosomes from the spindle (Schoborg *et al.* 2015). In this sense, Asp provides a true functional link between centrosome and spindle MTs whose functional interactions remain to be fully characterized.

Although perhaps mainly required for centrosome separation, Aurora A undoubtedly has other centrosomal functions by phosphorylating centrosomal and minus end-associated proteins. One such substrate is the MAP DTACC that, together with its partner Minispindles (Msps), fails to associate with spindle poles in the absence of Aurora A (Giet *et al.* 2002). A later study identified the Aurora A phosphorylation site in DTACC and confirmed that this phosphorylation was required to stabilize spindle MTs (Barros *et al.* 2005). Together, the DTACC:Msps complex appears to promote MT growth and stability at the mitotic centrosome, a function that has since been found to be highly conserved. The DTACC:Msps complex would thus appear to be another part of the crucial link between the PCM and spindle MTs *per se*. In this context, it is noteworthy that Zhang and Megraw (2007) have described a domain on the N-terminal part of Cnn required for the recruitment of not only γ-tubulin but also the DTACC:Msps complex.

## Centrosomes in Asymmetric Fate-Determining Cell Divisions

Cells generally divide symmetrically to replenish a tissue or increase its size. However, asymmetric cell division is important to generate daughter cells with different fates. The larval NBs in the brain and the ventral ganglion have been classically used to study asymmetric fate determining divisions. These cells divide asymmetrically to generate a ganglion mother cell (GMC), that enters a differentiation pathway and a self-renewed NB. GMCs then divide once more symmetrically to produce two terminally differentiated cells (neurons and/or glia) (Homem and Knoblich 2012). The establishment of cell polarity (Box 1) precedes mitotic spindle orientation for the subsequent asymmetric localization and differential segregation of cell fate determinants between the daughter cells.

Box 1 **Cell Polarization**

The apical Par (partitioning defective) complex, formed by Bazooka (Baz), Par-6, and atypical PKC (aPKC), establishes the polar axis and the basal localization of cell fate determinants such as Prospero (Pros), Brain Tumor (Brat), and Numb through their adaptor proteins Miranda (Mira) and Partner of Numb (Pon) (Homem and Knoblich 2012). The Pins [partner of Insc (Inscuteable)] complex also localizes apically and includes Insc, Pins, the heterotrimeric G protein subunit Gαi, and Mushroom body defect (Mud), and is mainly involved in aligning the spindle along the apicobasal axis through the interaction between Baz and Insc and between Mud and astral MTs. In mitosis, the Mud protein forms a crescent at the apical cortex and also localizes to the centrosome (Bowman *et al.* 2006; Izumi *et al.* 2006; Siller *et al.* 2006; Homem and Knoblich 2012). Pins is connected to the astral MTs also through a Mud-independent pathway involving kinesin Khc73 and Disc large (Dlg) and requiring Aurora A phosphorylation of Pins (Johnston *et al.* 2009).

**Figure 4:**
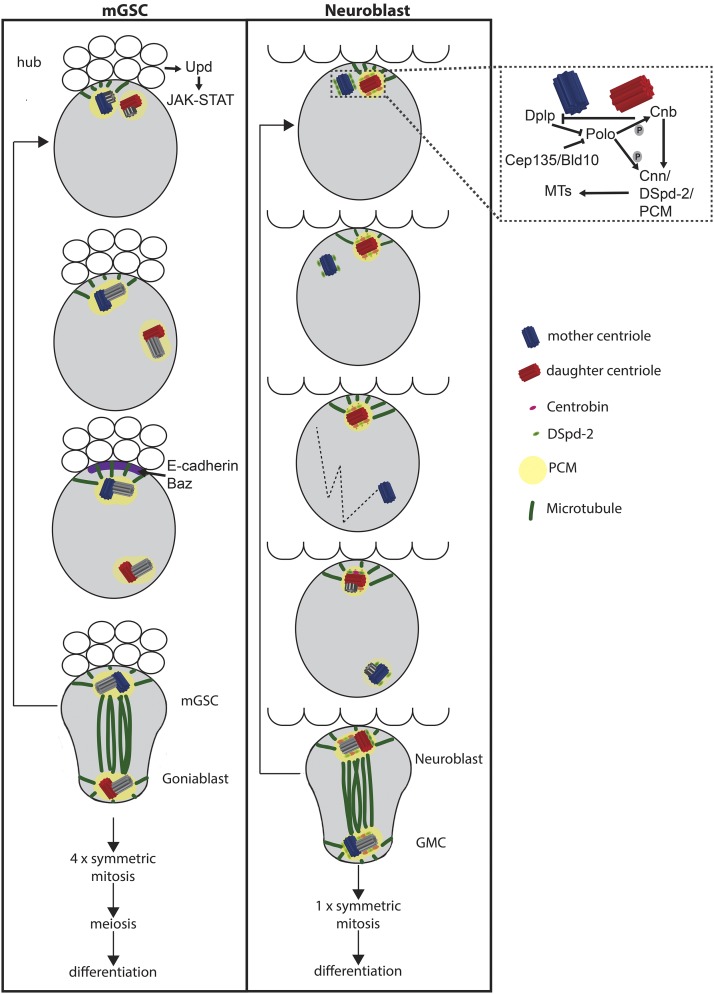
Centrosomes during asymmetric divisions. Comparison of centriole inheritance in asymmetric cell divisions. Neuroblasts (right) enter interphase with disengaged centrioles. The mother centriole loses PCM and start to migrate throughout the cytoplasm while the daughter centriole retains PCM and MT nucleation activity and localizes to the apical cortex. The interphase recruitment of PCM around the daughter centriole is mediated by Polo phosphorylation of Cnb and Cnn. Cep135/Bld10 facilitates the shedding of Polo from the mother centriole. As mitosis onset approaches, the mother centriole becomes stabilized at the basal side of the cell and accumulates PCM. It will be inherited by the ganglion mother cell. In mGSCs (left), when the duplicated centrosomes separate in G2 phase, the mother stays next to the hub whereas the daughter migrates to the opposite pole of the cell and is inherited by the goniablast. Cnb, Centrobin; Cnn, Centrosomin; GMC, ganglion mother cell; mGSC, male germ stem cells; MTs, microtubules; PCM, pericentriolar material.

All cortical markers disappear at the end of mitosis, except the apical centrosome that organizes the interphase MTOC and marks the position of the next apical crescent much earlier than the onset of asymmetric localization of any of the known markers of cortical polarity. This first suggested that this centrosome may have a role in transmitting information about spindle orientation from one cell cycle to the other (Rusan and Peifer 2007; Januschke and Gonzalez 2010). Centrosome splitting occurs before centriole duplication (Januschke *et al.* 2011) and the two centrosomes migrate together to the apical cortex. Later, the mother centrosome loses PCM and starts to migrate, initially within the apical side of the cell and more basally later on (Figure 4). As mitosis onset approaches, the mother centrosome becomes stabilized at the basal side and starts to accumulate PCM and organize the second aster. In this way, the spindle is assembled in alignment with the polarity axis of the cell and the mother centrosome is inherited by the differentiating GMC (Rebollo *et al.* 2007; Conduit and Raff 2010; Januschke *et al.* 2011). Polo kinase is the regulator of PCM recruitment (Glover 2005). Centrobin (Cnb) is a daughter centriole component important for PCM recruitment during interphase. This is achieved though Polo-mediated phosphorylation of both Cnb and Cnn, which allows the interphase recruitment of PCM around the daughter (Januschke *et al.* 2013). DPlp acts as an inhibitor of interphase PCM recruitment around the mother centrosome (Lerit and Rusan 2013). Another player is Wdr62, the fly homolog of WDR62/MCPH2 in vertebrates, that regulate neurogenesis in rat by controlling symmetric and asymmetric divisions and interacts with Aurora A kinase (Chen *et al.* 2014). Wdr62 maintains an active interphase MTOC by stabilizing MTs, which are necessary for recruitment of Polo to the PCM and downregulation of DPlp (Ramdas Nair *et al.* 2016).

Cep135/Bld10 has also been reported to play a role through shedding of Polo from the mother centrosome to establish asymmetry (Singh *et al.* 2014). Pins’s role in these events is not understood, but in its absence the daughter apical centrosome begins to behave like the mother, migrating across the cell during interphase and at mitosis spindle orientation is randomized (Rebollo *et al.* 2007).

Regulated loss of Polo kinase has also been recently described to precede the loss of PCM immediately before centrioles are eliminated from the female germline as nurse cells of the egg chamber undertake their endoreduplication cycles. Moreover, Polo-downregulation by RNAi *in vivo* accelerates centriole loss while ectopic tethering of Polo to centrioles prevents centriole loss (Pimenta-Marques *et al.* 2016). Thus, in this cell type, Polo appears to be required to maintain the very existence of the centrosome.

*Blastoderm-specific gene 25D* (*Bsg25D*) is the fly homolog of Ninein (*nin*), a centrosomal protein mutated in Seckel syndrome. In *Drosophila* NBs, Nin exhibits pericentrosomal localization and preferentially accumulates at the younger, daughter centrosomes in a cell cycle-dependent manner; Nin expression is only detected in interphase or early mitotic NBs and becomes undetectable at centrosomes by metaphase. Despite Nin’s role in mammalian neurogenesis, *nin^1^* homozygous flies are viable and fertile without any obvious phenotype, suggesting that in *Drosophila* this protein may have redundant function (Zheng *et al.* 2016).

Recently, the anaphase-promoting complex/cyclosome (APC/C) has been linked to mitotic exit and NB homeostasis through its interaction with Spd-2. In particular, in interphase, Spd-2 acts as the centrosomal linker for the recruitment of the interphase APC/C activator Fizzy-related (Fzr), maybe allowing the APC/C to efficiently target its centrosomal substrates to couple the centrosome function to cell cycle progression (Meghini *et al.* 2016).

Centrosome asymmetry also directs asymmetric cell division in the *Drosophila* male germline (Figure 4; Rebollo *et al.* 2007; Yamashita *et al.* 2007). *Drosophila* mGSCs are active throughout adulthood to ensure the production of gametes and are associated with the hub cells, which constitute the stem cell niche. mGSCs normally divide asymmetrically with the spindle oriented perpendicular to the hub, producing one stem cell, which remains associated with the hub, and one goniablast, which initiates differentiation. So far, no cell fate determinant has been identified during asymmetric cell division in mGSCs and it is believed that the microenvironment provides information for cell fate specification. In G_1_, the single centrosome is located near the interface with the hub. When the duplicated centrosomes separate in G_2_, the mother stays next to the hub, whereas in contrast to NBs, the daughter migrates to the opposite side of the cell and is inherited by the goniablast (Yamashita *et al.* 2003, 2007). Centrosome positioning requires Cnn, Apc2 (Yamashita *et al.* 2003), and E-cadherin-based adherens junctions (Inaba *et al.* 2010). If the centrosomes of mGSCs are misoriented, cells become arrested or delayed in the cell cycle (Cheng *et al.* 2008) and Cnn, Par-1, and Baz/Par-3 (a substrate of Par-1) are critical components of this centrosome orientation checkpoint (COC). Baz forms a subcellular structure (Baz patch) at the hub-GSC interface in an E-cadherin-dependent manner, this patch anchors the apical centrosome prior to mitotic entry, and centrosome-docked Baz inactivates COC (Inaba *et al.* 2010, 2015; Yuan *et al.* 2012).

A different situation pertains in the *Drosophila* ovary, where daughter centrosomes are preferentially inherited by the female GSC (fGSC) (Salzmann *et al.* 2014). Here, the two centrosomes appear to be randomly positioned within the cell and it is only after mitotic spindle formation that centrosomes orient relative to the spectrosome and niche. Analysis of *DSas-4* mutant stem cells suggests that centrosomes are not required for the proper orientation of the spindle relative to the spectrosome or niche in fGSCs, or for the proper orientation of the spindles relative to the fusome in mitotic cysts (Stevens *et al.* 2007).

When cells from larval brains of mutants in genes involved in cell fate determination in the asymmetric divisions of NBs (Box 1) are implanted into the abdomens of wild-type adult hosts, they develop cancer-like masses of cells that show genome instability, centrosome alterations, and metastasis (Caussinus and Gonzalez 2005). Subsequently, the systematic use of transplantation to analyze the tumorigenic potential of well-characterized mutants of genes required for centriole and centrosome function revealed that *asl*, *Plk4*, *Sas-6*, *Sas-4*, *polo*, and *aurA* mutant larval brain tissue all generated tumors in the abdominal cavity that in several cases led to metastasis. Allograft cultures of imaginal discs (that divide symmetrically) taken from the same mutants were not able to induce tumors. These results led the authors to propose that different types of centrosome-related dysfunction can trigger tumorigenesis by compromising the self-renewing asymmetric divisions (Castellanos *et al.* 2008). Genomic instability is unlikely to lie behind tumor formation by NBs with centrosomal defects because mutants that give aneuploidy in the absence of centrosome dysfunction do not give rise to tumors in this system (Castellanos *et al.* 2008). Moreover, NBs overexpressing Plk4 have predominantly bipolar spindles in larval NBs because of centrosome clustering and so chromosome segregation is not dramatically affected, and yet these cells are tumorigenic in the transplantation assay (Basto *et al.* 2008). Taken together, the most likely explanation for these findings is that defects in centrosome function perturb the inheritance of cell fate determinants and it is this that causes tumor growth (Gonzalez 2007; Januschke and Gonzalez 2008).

The findings that different mechanisms govern the differential behavior of centrioles/centrosomes in asymmetric divisions in different tissues of the same organism point to the importance of carrying out studies in the whole organism. It is a cautionary tale of the importance of not drawing generalized conclusions based upon findings in just a single cell type.

## When the Centriole Becomes the Basal Body: Ciliogenesis

The centriole has a dual life that is perhaps nowhere more evident than in mammalian cells which, when they stop cycling and enter a quiescent G_0_ phase, generate a structure known as the primary cilium. In this process, the distal end of the mother centriole associates with the ciliary vesicle and proceeds to dock with the plasma membrane to become a basal body. This structure templates cilium formation, which requires the intraflagellar transport system to elongate the ciliary axoneme [reviewed by Pedersen and Rosenbaum (2008), Ishikawa and Marshall (2011), and Avasthi and Marshall (2012)].

The primary cilia have particular importance in mammalian cells as they serve as a center for several signaling cassettes that play essential roles in the development and physiological function of tissues. In *Drosophila*, cilia are only present in a small number of cell types that have specialized functions.

### Ciliated sensory neurons

**Figure 5:**
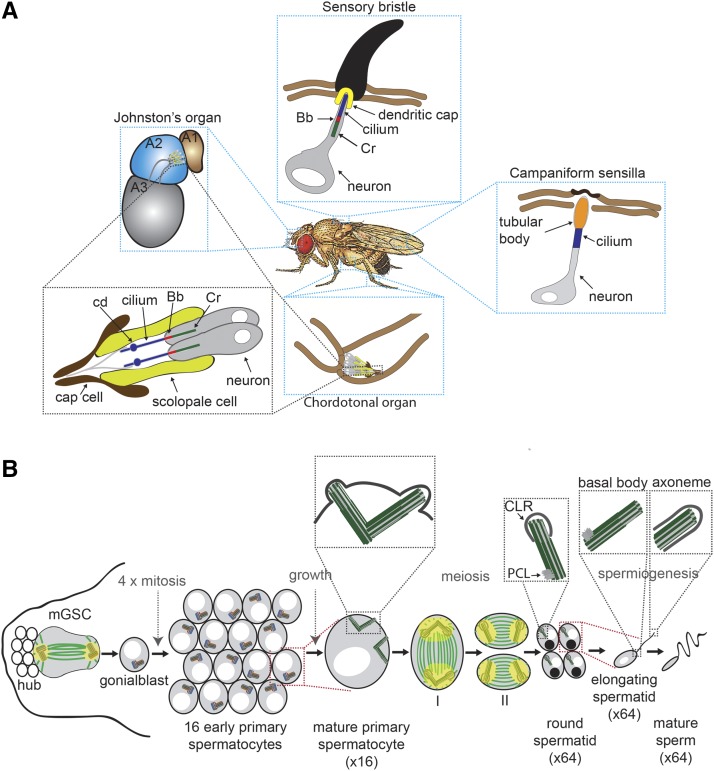
The cilia and flagellae in sensory organs and in the male germline of *Drosophila*. (A) Examples of different ciliated tissues in *Drosophila* adult and schematic representation of ciliated neurons. (B) Overview of different stages of spermatogenesis from asymmetric mGSC division to sperm differentiation. A mGSC divides asymmetrically to generate another mGSC and a goniablast. The goniablast divides mitotically four times to give rise to a cyst of 16 early primary spermatocytes. Primary spermatocytes then enter in a growth phase during which cell size increases and centrioles migrate to the cell surface where they assemble the primary cilium. At the beginning of meiotic division, centrioles move close to the nucleus with their associated CLR and membrane. The CLR is present in all four centrioles and is maintained during the meiotic divisions. At the end of meiosis, each primary spermatocyte generates four haploid round spermatids each containing a single centriole. At this stage, a small PCL is present at the proximal end of the centrioles. Round spermatids then differentiate into elongated sperms and centrioles become the basal bodies of the sperm axoneme. Details show the structure of centrioles. Components are not to scale. Bb, basal body; Cd, ciliary dilation; CLR, cilium-like region; Cr, ciliary rootlet; mGSC, male germ stem cells; PCL, procentriole-like structure.

This restricted number of *Drosophila* cells carrying cilia include spermatocytes (below) and the type I monodendritic sensory neurons of the peripheral nervous system. Two types of sense organ have such sensory neurons: external sense (ES) organs responsible for mechano-, chemo-, or olfactory-sensory functions, and chordotonal organs responsible for proprioception and hearing (Figure 5A) (Kernan 2007; Keil 2012; Vieillard *et al.* 2015; Jana *et al.* 2016). The cilia of type I neurons characteristically lack the central MT doublet. The proximal axonemal part harbors dynein arms that serve to amplify vibrational input. The distal part lacks dynein arms and has the receptor ion channels required for sensing. The axonemes of cilia in ES organs are devoid of dynein arms and show specialized distal outer segments [for review see Keil (2012)].

Basal bodies are situated at the base of the cilia from which they are separated by a short transition zone (TZ). Neurons of the chordotonal organs or olfactory sensilla have a tandemly arranged pair of centrioles at the apical tip of the sensory dendrite. The most distal centriole serves as the basal body from which the axoneme projects and, despite its lack of distal appendages, is recognizable as the mother because it has most of the DPlp. In contrast, the presence of Centrobin (Cnb) marks the proximal daughter centriole (Galletta *et al.* 2014; Gottardo *et al.* 2015a). Strikingly, the daughter becomes able to form cilia-like structures upon loss of Centrobin. Conversely, forced localization of Centrobin to the mother completely suppresses cilia formation (Gottardo *et al.* 2015a).

Centriole components are essential to build basal bodies and hence sensory cilia. Consequently, a common characteristic feature of mutants of genes for centrosomal proteins is severe lack of coordination as a result of the loss or abnormal function of cilia on femoral chordotonal organs. Thus, flies mutant in the Zone III component DPlp are severely uncoordinated and show defects in mechanosensory neuron function, and cilia with centrioles were located within the cell bodies and not near the dendrite tip (Martinez-Campos *et al.* 2004; Galletta *et al.* 2014). Interestingly, the function of mechanosensory neurons relies on DPlp’s interaction with calmodulin whereas basal body formation in the testis does not (Galletta *et al.* 2014). Severe lack of coordination associated with early death and cilium loss were reported also for mutants of the centriolar components Sas-4 and Sas-6 (Basto *et al.* 2006; Peel *et al.* 2007).

The ciliary rootlet of sensory neurons connects the cell body to basal bodies. It is variable in length depending on the neuron type but interestingly this structure is not found in testis (Jana *et al.* 2016). The ciliary rootlet is comprised of striated fibers, it assembles on basal bodies, and extends toward the cell body. Rootletin is the major components of rootlets in *Drosophila* chordotonal and external sensory neurons and it is indispensable for rootlet but not for cilium assembly, thus is consequently necessary for neuronal sensory responses (Chen *et al.* 2015; Styczynska-Soczka and Jarman 2015). Rootlet formation and the cohesion of basal bodies are disrupted in *rootletin* mutants; however, the cilium structure appears to be normal. Mutations in centriolar components but not in PCM components impair rootlet formation. Thus, Rootletin directs rootlet assembly in a centriole-dependent but cilium-independent fashion. Interestingly, Cep135/Bld10 is required for localization of ectopically-expressed Rootletin to brain centrosomes and testis basal bodies. However, it is dispensable for Rootletin assembly in ciliated neurons, highlighting another particularity of the basal body organization in type I neurons (Chen *et al.* 2015).

### Centrioles and basal bodies during spermatogenesis

Spermatogenesis can be divided into three phases: first, a mitotic phase when mGSCs divide to generate another mGSC and a goniablast which will divide mitotically four times to create a cyst of 16 primary spermatocytes; second, a meiotic phase when primary spermatocytes divide synchronously to produce 64 haploid spermatids; and third, a final differentiation phase of the round haploid spermatids into spermatozoa (Figure 5B). The *Drosophila* male meiosis I spindle is some 23.3 ± 0.2 µm long compared to a NB mitotic spindle of 11.7 ± 3.3 µm. It has been proposed that these huge spindles of the primary spermatocytes provide effective stores of tubulin needed for subsequent sperm tail formation (Lattao *et al.* 2012). Centrosomes and centrioles are essential for the assembly of these spindles (Bonaccorsi *et al.* 1998; Rebollo *et al.* 2004; Giansanti *et al.* 2008) and axoneme formation during spermiogenesis (Bettencourt-Dias *et al.* 2005), respectively.

Centrioles undergo dramatic changes in size and morphology during spermatogenesis (Tates 1971). Centrioles in young primary spermatocytes are 0.9 µm in length, with a cartwheel and a proximal part of nine triplets, a central tubule and a distal part with no central tubule, and the nine peripheral structures having one closed and one open tubule. The centriole maintains the cartwheel throughout spermatogenesis (Riparbelli *et al.* 2012). A short “cilium-like region” (CLR) (Riparbelli *et al.* 2012) projects from the cell membrane. Primary spermatocytes enter a growth phase of ∼90 hr in which centrioles elongate to ∼2.3 µm by the time of the metaphase of meiosis I (Tates 1971).

Centriole pairs dislodge from the plasma membrane at the onset of meiosis and move into the cytoplasm to nucleate astral MTs. The primary cilium persists at the distal end of centrioles and is associated with a “membrane pocket” or “ciliary pocket-like” structure as a basal body–cilium complex (Tates 1971; Riparbelli *et al.* 2012). The anatomy of this structure is described elsewhere (Gottardo *et al.* 2013).

At the end of meiosis, each spermatid inherits one centriole–CLR complex that will become the basal body and the sperm flagellum. Before the spermatids begin to elongate, there is an outgrowth of the distal part of the centriole, which is about twice as long as the original distal part of the centriole. The centriole region has triplets of MTs in a 9 + 1 arrangement and, at the transition zone between centriole and CLR, there is a gradual reduction of C-tubule protofilaments in the transition from centriolar triplets to axonemal doublets. Toward the tip of the CLR, the B-tubule is reduced and only the A-tubule is present. At this stage, the CLR lacks a central pair (Gottardo *et al.* 2013). It has been shown that *bld10* mutant spermatocytes have shorter centrioles, premature centriole disengagement, and that their axonemes are missing the central MT pair (Mottier-Pavie and Megraw 2009; Carvalho-Santos *et al.* 2010, 2012).

Close to the basal body of spermatids, there is a small disk of dense material, the “proximal centriole-like” or PCL (Blachon *et al.* 2009; Gottardo *et al.* 2015c), that may represent the origin for the second centriole in the *Drosophila* zygote (Blachon *et al.* 2014; Khire *et al.* 2016). PCL formation depends on Plk4, Sas-6, and Poc1, and the PCL contains many centriolar proteins that include Sas-6, Ana1, and Bld10p/Cep135 (Blachon *et al.* 2009).

In elongating spermatids, the proximal part of the centriole becomes the proper “basal body” and can be divided into a caudal part, which is continuous with the axoneme and lies outside the nucleus, and an apical part with an apparent intranuclear location. The caudal part is surrounded by a cylinder of an electron-dense structure called the “centriole adjunct” with a length of ∼1.10 µm, an outside diameter of ∼0.45 µm, and a wall thickness of ∼0.10 µm. During elongation, the centriole adjunct becomes shorter and wider and, in late spermatids, the centriole adjunct is no longer present (Tates 1971). Axoneme elongation is a coordinated process in which the distal end of the axoneme is enclosed in the ciliary membrane and the axoneme is surrounded by ER membranes and the two giant mitochondrial derivatives of the Nebenkern [reviewed in Fabian and Brill (2012)].

The TZ between the basal body and the ciliary membrane plays both structural and functional roles by regulating the traffic to and from, and by forming structural links between, MTs and the membrane both in the elongating axoneme and in the primary cilium of spermatocytes (Vieillard *et al.* 2016). In mammals and *C. elegans*, three protein complexes (MKS, NPHP, and CEP290) play key roles in TZ assembly. *Drosophila* is lacking NPHP components but the CEP290 and most MKS module proteins are present together with two other proteins, Chibby (Cby) and Dilatatory (Dila), which localize at the TZ and are involved in cilia assembly (Ma and Jarman 2011; Enjolras *et al.* 2012; Basiri *et al.* 2014). All the TZ proteins form a “ciliary gate” or “ring centriole” that continuously migrates away from the centriole to compartmentalize the growing axoneme tip (Fabian and Brill 2012; Basiri *et al.* 2014). In spermatocytes, Cby colocalizes with acetylated tubulin and Cep290 is found in an overlapping yet distinct inner region of the TZ that also overlaps with axonemal MTs (Pratt *et al.* 2016). Uncoordinated (Unc) marks the whole centriole and cilium/TZ in spermatocytes (Baker *et al.* 2004). Cby and Unc colocalize along the TZ/cilia, but Cby extends a little more distally to Unc and Unc distribution seems to be dependent on Cby (Enjolras *et al.* 2012). In *unc* mutants, spermatid nuclei detach from basal bodies and flagellar axonemes are disrupted (Baker *et al.* 2004). This reflects a role in the remodeling of the TZ at the onset of flagella elongation: Cby and a ring of Unc separate from the basal body; MKS components become redistributed along an extended membrane cap; and Dila is maintained, like Unc, at the basal body and a little at the centriole but is disassembled from both the basal body and TZ soon after initiation of spermatid elongation. Cby, MKS, and Unc are all disassembled from the ring centriole as sperm begin to individualize (Vieillard *et al.* 2016).

During spermiogenesis, levels of many centrosomal component proteins become diminished in a process known as centrosome reduction. Asl protein is one such example, Asl has a role in sperm basal body function by ensuring its attachment to the spermatid nucleus as well as in the assembly and/or maintenance of the flagellar axoneme (Galletta *et al.* 2016a). However, although present along the length of the centriole in round spermatids, Asl shrinks to occupy a collar-like structure during spermatid differentiation. Later, Asl is only detectable in the PCL and at the proximal end of the centriole. Finally, in spermatozoa, Asl is barely detectable. Asl reduction is regulated by Plk4 and Slimb, and attenuation of Asl reduction causes delayed development and a failure to form astral MTs in the zygote suggesting that centrosome reduction is essential for postfertilization development (Khire *et al.* 2015).

## Concluding Remarks

Centrioles play a key role in the development of the fly. They are needed for the correct formation of centrosomes, essential to give organization to the rapidly increasing numbers of nuclei in the syncytial embryo, and for the spatially precise execution of cell division in numerous tissues, particularly during male meiosis. Although mitotic cell cycles can take place in the absence of centrosomes, this is an error-prone process that opens the fly to developmental defects and the potential of tumor formation. The functions of the centriole in its other life, as a basal body, do not seem to be as widespread in the fly; unlike in mammals, where the great majority of cells appear to have primary cilia that function in signal transduction pathways, the fly has cilia restricted to specialized cell types of the neurosensory organs and the male germline. That said, it is noteworthy that the *unc* gene product, involved in the assembly of the ciliary axoneme and therefore expected to be found only in type 1 sensory neurons and male germ cells, is present in evenly spaced spots in the larval eye disc in association with mother centrioles in the R8 photoreceptor cell (Gottardo *et al.* 2016). The biological meaning of this observation is uncertain and it remains possible that it represents a vestige of a ciliary function that has been lost during evolution. However, it is a pointer to the many unknowns that remain in studying centriole, basal body, and ciliary function in the fly. It is evident from our discussion above that there are both differences in ciliary architecture between different cell types and different requirements upon the centrosome at different developmental stages. *Drosophila* is an excellent organism in which to explore such potential tissue and developmental stage differences in organelle function, and this is an area in which we can expect some interesting findings to emerge in future years.
